# A stochastic model of randomly accelerated walkers for human mobility

**DOI:** 10.1038/ncomms12600

**Published:** 2016-08-30

**Authors:** Riccardo Gallotti, Armando Bazzani, Sandro Rambaldi, Marc Barthelemy

**Affiliations:** 1 Institut de Physique Théorique, CEA, CNRS-URA 2306, F-91191 Gif-sur-Yvette, France; 2 Department of Physics and Astronomy, University of Bologna, Viale Berti Pichat 6/2, 40126 Bologna, Italy; 3 INFN Bologna Section, 40126 Bologna, Italy; 4 CAMS (CNRS/EHESS) 190-198, avenue de France, 75244 Paris, France

## Abstract

Recent studies of human mobility largely focus on displacements patterns and power law fits of empirical long-tailed distributions of distances are usually associated to scale-free superdiffusive random walks called Lévy flights. However, drawing conclusions about a complex system from a fit, without any further knowledge of the underlying dynamics, might lead to erroneous interpretations. Here we show, on the basis of a data set describing the trajectories of 780,000 private vehicles in Italy, that the Lévy flight model cannot explain the behaviour of travel times and speeds. We therefore introduce a class of accelerated random walks, validated by empirical observations, where the velocity changes due to acceleration kicks at random times. Combining this mechanism with an exponentially decaying distribution of travel times leads to a short-tailed distribution of distances which could indeed be mistaken with a truncated power law. These results illustrate the limits of purely descriptive models and provide a mechanistic view of mobility.

## Abstract (web summary)

Many human mobility studies have shown empirically long-tailed distance distributions, which are usually associated to Lévy flights. Here, the authors show that the behavior of private vehicles could be misinterpreted as Lévy flights but is fully captured by a class of accelerated random walks.

## Graphical abstract

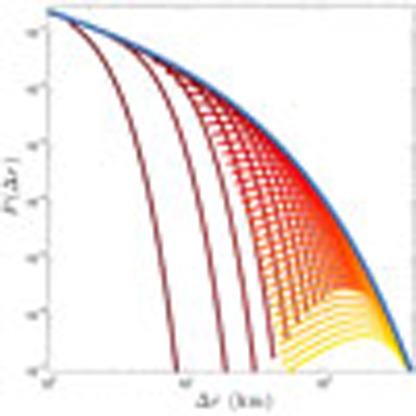

Understanding individual mobility has important implications for traffic forecasting^1^, epidemics spreading^2^,^3^ or the evolution of cities^4^,^5^,^6^. With the development of Information and Communication Technologies^7^, the investigations' focus shifted from the traditional travel diary surveys^8^,^9^,^10^ to several new data sources. In particular, it became possible to follow individual trajectories from mobile phone calls^11^,^12^,^13^, location-sharing services^14^,^15^,^16^ and microblogging^17^, or directly extracted from public transport ticketing system^10^,^18^, global positioning system (GPS) tracks of taxis^10^,^19^,^20^,^21^,^22^,^23^, private cars^24^,^25^,^26^ or single individuals^27^,^28^. For most data sources, the spatial position *r* is the most reliable quantity. This information can be used for studying two different aspects of human mobility: how far and where we are moving. The second question is far more complex than the first and can be approached with several different tools, from aggregated origin–destination matrices^29^ for mobility prediction^30^ or land use analysis^31^ to individual mobility networks and patterns suitable for describing the natural tendency to return frequently to a few locations (such as homes, offices and so on)^11^,^12^,^24^,^25^,^26^,^32^. On the other hand, the first question, generally characterized by the distribution *P*(Δ*r*) of the individuals' displacements Δ*r* across all users, although apparently simple is still far from being completely understood. Indeed, even if the study of the distribution *P*(Δ*r*) has become a trademark for recent works on human mobility, there are still no consensus about the functional form of this distribution. At a large scale (national or inter-urban), one may observe a long tail behaviour^9^,^11^,^12^,^14^,^15^,^17^,^20^,^22^,^33^ characterized by a power law decay for long displacements. At a smaller (urban) scale, the distribution seems to have an exponential tail^10^,^13^,^19^,^21^,^22^,^24^,^25^. The unclear nature of this probability distribution makes its interpretation difficult and dependent on the data set used, the scale and possible empirical and fitting errors^34^. It is therefore necessary to obtain data as clean as possible and to propose a model that can be tested against empirical results. So far, essentially power law fits were used and led the authors to draw conclusions about the nature and mechanisms of the mobility, but this way of proceeding could actually lead to erroneous conclusions. Similar unresolved controversy also exists in the study of animal's foraging movements: relying only on a fit of the empirical data, the same distribution can be understood in different ways, leading to contrasting conclusions on the nature of the underlying process^35^, ^36^, ^37^, ^38^.

Remarkably enough, what appears to be under-evaluated in the study of human mobility is the relevance of travel itself. Human travelling behaviour can in general be described as a sequence of rest times of duration 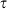 and jumps Δ*r* in space^12^. These two processes need to be separated for modelling human mobility, since costs are in general associated to trips while a positive utility can be associated to activities performed during stops^1^. However, proposed models usually neglect the role of travel time and the moving velocity and assume instantaneous jumps. This is essentially a consequence of the limitations inherent to data sources: phone calls or social networks capture the spatial character of individuals' movements^39^, but are limited by sampling rates or by the bursty nature of human communications^40^,^41^ and are thus not suitable for an exhaustive temporal description of human mobility.

In this paper, we show that the observed truncated power laws in the jump size distribution can be the consequence of simple processes such as random walks with random velocities^42^. We test this model over a large GPS database describing the mobility of 780,000 private vehicles in Italy, where travels and pauses can be easily separated, as the transition is identified by the moment when the engine is turned on or off (but we introduce a lower threshold of 5 min in the elapsed time, to distinguish real stops from accidentally switched off of the engine during a trip). This allows us to evaluate accurately not only the displacements Δ*r*, but also travel times *t*, speeds *v* and rest times 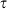.

## Results

### The current empirical view

**Figure 1:**
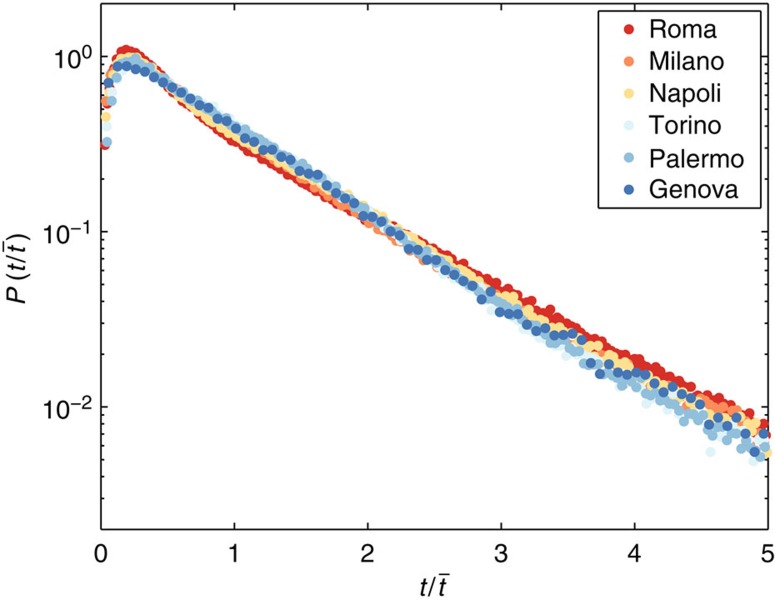
Travel time distribution in the six largest Italian cities. The probability distribution *P*(*t*) is well fitted by an exponential function. As demonstrated by the data collapse shown here, differences among cities are encoded in a single parameter: the average travel time 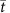.

Several studies suggested that the displacements' distribution *P*(Δ*r*) has a fat tail, and power law fits display a wide range of exponent values depending on the data set and the fitting form used (see Table 1). We note that, strictly speaking, the distribution cannot be scale-free since displacements are always limited in space^43^. Thus, human movements could possibly be identified as truncated Lévy flights only^11^,^44^. In contrast, displacements at the urban scale consistently display an exponential tail^19^,^24^,^25^. Short tails also emerge when studying the distribution *P*(*t*) of travel times *t* of individual trajectories originating in different cities. For private cars' mobility, as in the data set studied here, we observe that *P*(*t*) is indeed characterized by an exponential decay 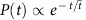 as in refs 9, 19, 28, 45 (see Fig. 1), where 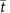 is the average travel time that may vary among cities (see Supplementary Note 1 and Supplementary Fig. 1). For public transportation, we also observe a rapidly decreasing tail for the travel times between metro stations (see ref. 18 and Supplementary Fig. 2). A short tail is expected for these distributions, since the total daily travel time spent in public^46^ or private transportation^45^ has an exponential tail (see Supplementary Note 1 and Supplementary Fig. 3 for the analysis of the distribution *P*(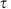) of rest times 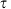).

We have therefore ambiguous empirical results and purely descriptive models, leading to a very unclear view of human mobility. Here we investigate the statistics of displacements starting from simple assumptions about the dynamics that governs mobility. In contrast with empirical and descriptive approaches, we start by modelling this mechanism and show that our predictions are consistent with data. To model mobility, we must understand the relationship between the duration of a trip and its average velocity. Velocity is a natural quantity for describing the mobility, and classical traffic modelling focuses on predicting its average value in freeways or in cities with different vehicle's densities^47^. The relevance of velocity appears to be underestimated in recent studies even when the hierarchy of transportation networks is suggested to be at the origin of the fat tail^48^. As discussed above, this omission is a consequence of data sources limitations. To go beyond descriptive approaches, it is necessary to obtain richer data such as the large GPS database used here.

### Random uncorrelated accelerations

**Figure 2:**
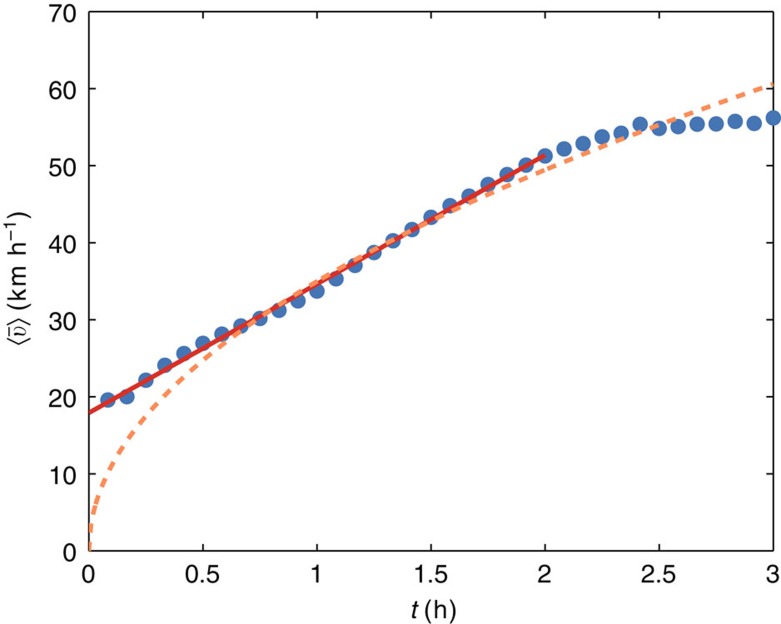
Acceleration of private transportation. Empirical average speeds (blue dots) versus the duration of the trip for our GPS data set. The red solid line represents a constant average acceleration 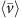
=*v*_0_+*at* with *v*_0_=17.9 km h^−1^ and *a*=16.7 km h^−2^. For *t*>2 h the speed reaches a saturation at 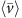
≈55 km h^−1^ imposed by the finite number of layers (see Methods section and Supplementary information). The dashed line represents the best fit with 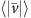
∝*t*^1/2^ (as predicted by the simple random model equation (1) with uncorrelated accelerations).

A first remark is that apparent truncated power laws can result from interrupting at random times simple processes^49^ such as random walks in the velocity space. To illustrate the problem with this simple approach, we consider the evolution of the velocity *v* described by a Brownian motion with diffusion coefficient *D*


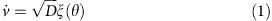


where *θ* is time and *ξ* is a white noise. This random acceleration model has been the subject of many theoretical studies (see ref. 50 and references therein) and provides here an interesting null model. We define the displacement for a given time time *t* as 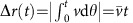 where >0 is the average velocity. To compute the displacement distribution, we use the fact that 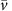 is a Gaussian variable and that the travel time distribution is approximatively exponential 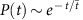. Using a saddle-point approximation (see Supplementary methods), we obtain a stretched exponential distribution for large displacements


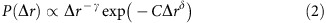


with *γ*=3/4, *δ*=1/2 and *C* a free parameter. We show in Supplementary Fig. 4(a) the best fit with *C*=0.49 km^−0.5^. At this stage, this model offers an already reasonable description of the empirical pattern usually described by a truncated power law with three parameters^11^ which results here from the combination of a random walk and a random duration model. In addition, this simple random model (equation (1)) implies a relation^51^ between the travel time *t* and the average velocity modulus of the form 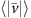
∝*t*^1/2^. However, for both private cars (see Fig. 2) and public transportation (see Supplementary Fig. 5(a) and Supplementary Note 2), we find that average speed grows linearly with the travel time. This important empirical observation invalidates our first simple model (which might still be relevant for describing different mobility patterns, such those of animals^52^) and one has to understand the origin of this uniform acceleration. Our idea here is that this behaviour results from the optimal use of the hierarchical nature of the transportation networks for longer trips. Indeed, it is likely that faster transportation modes or faster roads are used more frequently for longer trajectories^25^,^53^. In the following, we propose a stochastic model based on this idea, which correctly predicts both travel speed and displacement distributions.

### Random acceleration kicks

**Figure 3:**
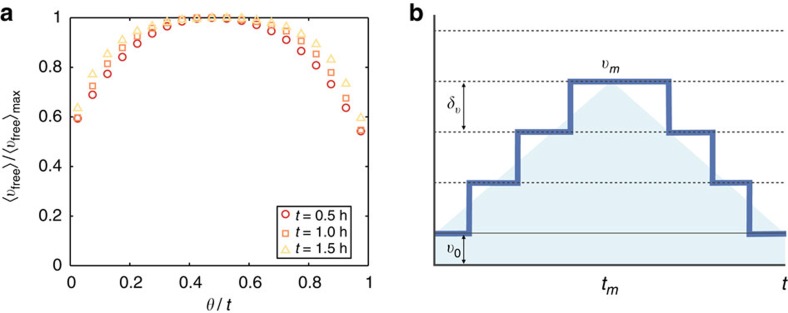
Empirical evidence and schematic representation of the model. (a) We compute for different trips the empirical average free-flow speeds 〈*v*_free_〉 for the used roads, measured in the absence of congestion (see Methods section). We then aggregate the trips according to their duration *t*, and we plot the evolution of the relative average free-flow speed 〈*v*_free_〉/〈*v*_free_〉_max_, where 〈*v*_free_〉_max_ is the maximal average free-flow speed reached at *θ*≈*t*/2. We observe symmetrical curves with a progressive increase of velocity in the first half of the trip, a short flat central part followed by a deceleration. The curves do not collapse, since longer trips have a larger fraction of their trajectory spent at a maximal speed. The maximal speed 〈*v*_free_〉_max_ is reached at the middle of the trip and increases from 79 km h^−1^ for trips with a duration *t*=0.5 h to 106 km h^−1^ for a duration of *t*=1.5 h. (**b**) Schematic representation of the random acceleration model (equation (3)). Each trajectory starts with an acceleration phase, where the speed increases by constant kicks equal to *δv*. These kicks happen at random times with an uniform probability per unit time *p*. When approaching the destination, there is a deceleration phase with random kicks of −*δv* (with the same probability *p*). The symmetry of the problem implies that the maximal speed *v*_*m*_ is reached on average at *t*_*m*_=*t*/2 and depends on *δv* and *p*: *v*_*m*_=*v*_0_+*k*(*t*_*m*_)*δv*, where *k*(*θ*) is the number of kicks after a time *θ* and follows a Poisson distribution of average 〈*k*(*θ*)〉=*pθ*. The average speed 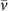 of the trajectory is evaluated by estimating the shaded area and leads to 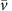
=(*v*_0_+*v*_*m*_)/2.

In contrast with the assumption underlying equation (2), accelerations are not uncorrelated: in Fig. 3a, we indeed observe that an average trip can be separated into a first half where one uses progressively faster roads and a second half with deceleration until the velocity reaches back the base speed at time *t*. We use this simple representation in a stochastic model (see the schematic in Fig. 3b) and make the following assumptions. The transportation network is modelled by *n* layers *L*_*n*_, corresponding to different travel speeds *v*_*n*_ (we neglect saturation effects, see Methods section and Supplementary methods). The speed differences between layers are taken constant and equal to *δv*, and the speed on layer *L*_*k*_ is then *v*_*k*_=*v*_0_+*kδv*. An individual starts then her trip of duration *t* in the layer *L*_0_ with base speed *v*_0_, and we assume that there are two phases in a trip, acceleration and deceleration, of roughly the same duration. In both the ascending and descending phases, we define a Poissonian process where all individuals have the same probability per unit time *p* to jump to the successive layer and to change their speed.

**Figure 4:**
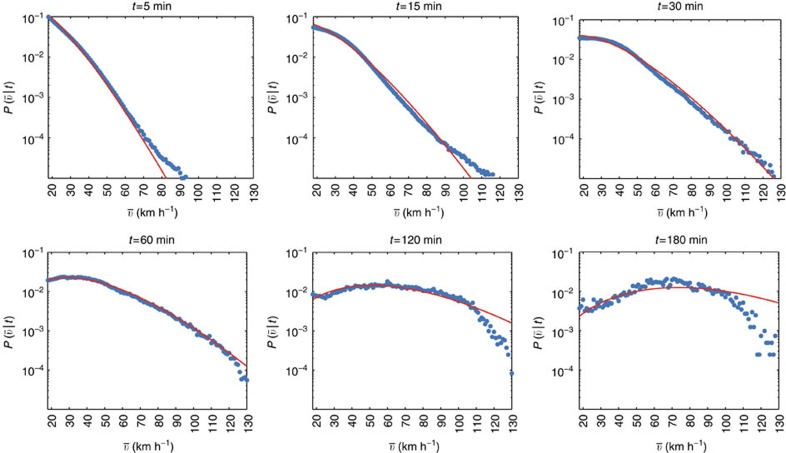
Conditional distribution 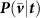 of the average speeds for different travel times. We fit simultaneously the curves 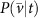 given by equation (4) in the interval [*v*_0_, *v*_max_], with *v*_0_=17.9 km h^−1^ and *v*_max_=130 km h^−1^ and for *t*=5, 10, 15, 20, …, 180 min (see Methods section). Each plot refers to a different trip durations (*t*=5, 15, 30, 60, 120, 180 min). The dots are the empirical data whereas the solid line is the fit obtained using equation (4). The best fit value of the parameters are *p*′=1.06 jumps per hour, *δv*′=20.9 km h^−1^ (and *v*_0_=17.9 km h^−1^). We therefore get *δv*≈40 km h^−1^ for the speed gap, in excellent agreement with the progression of the most common speed limits in Italy: 50 km h^−1^ (urban), 90 km h^−1^ (extra-urban) and 130 km h^−1^ (highways). These results suggest that a multilayer hierarchical transportation infrastructure can explain the constant acceleration observed in both public and private transportation.

**Figure 5:**
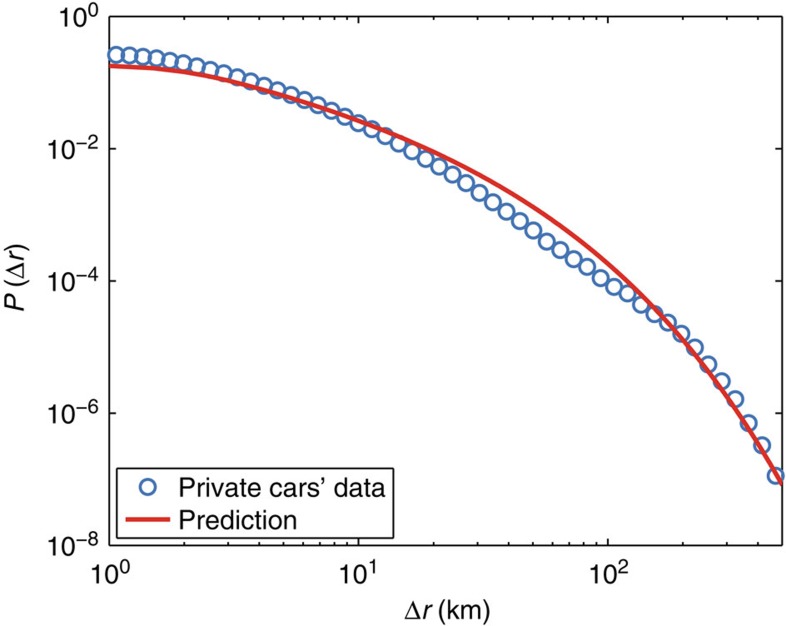
Displacements distribution. We show (circles) the aggregated empirical distribution *P*(Δ*r*) for all the ≈780,000 cars in the data set. The solid line is the model prediction (and not an *a posteriori* best fit) based on our model equation (5) with: (i) *v*_0_=17.9 km h^−1^ estimated as the intercept of the fit in Fig. 2; (ii) *p*′=1.06 jumps per hour and *δv*′=20.9 km h^−1^ estimated from the fit of 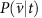 in Fig. 4; and (iii) 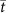
=0.30 h coming from the average of the travel times for all selected trips (see Methods section). This remarkable prediction has a quality comparable with the commonly used direct fits by a truncated power law with three free parameters (see Supplementary Fig. 4).

Within this model, we can estimate the maximal speed *v*_*m*_=*v*_0_+*k*(*t*_*m*_)*δv*, where *k*(*t*_*m*_) is the number of jumps at mid trajectory *t*_*m*_=*t*/2, and get approximatively the average speed 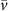
≈(*v*_0_+*v*_*m*_)/2. Since the process is Poissonian, we have 〈*k*(*t*_*m*_)〉=*pt*_*m*_ and the average speed is given by


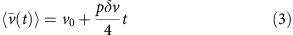


where the brackets denote the average over the Poisson variable *k*. The average speed thus grows linearly with *t*, in agreement with empirical observations. Remarkably enough, this model allows us to predict also the shape of the conditional probability distribution 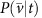. Indeed, the number of jumps *k* is distributed following the Poisson distribution 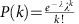 with *λ*=*pt*/2. Using the Gamma function as the natural analytic continuation of the factorial *k*!=Γ(1+*k*), we obtain the distribution


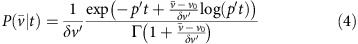


where *p*′=*p*/2 and *δv*′=*δv*/2 are free parameters fitted using empirical speeds (see Fig. 4). The shape of the displacement distribution *P*(Δ*r*) can then be computed as a superimposition of Poisson distributions (see Supplementary Fig. 4(b)) and is given by


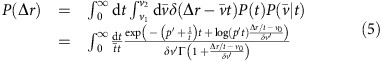


where *δ*(*x*) is the Dirac delta function. This equation (5) is our main analytical result and its exact form cannot be exactly computed, but the limiting behaviour for large Δ*r* is again equation (2) (see Supplementary methods). In particular, this distribution is not fat-tailed, in clear contrast with Lévy flights which have divergent moments and are governed by large fluctuations. Therefore, all phenomena associated to Lévy flights such as superdiffusion, for example, are not expected from our model. In Fig. 5, we compare the empirical *P*(Δ*r*) with our prediction and show that the proposed random acceleration model (together with the exponential behaviour for *P*(*t*)) is in excellent agreement with data.

## Discussion

The identification of an approximate power law behaviour of a complex system is rarely scientifically useful by itself^43^ but needs to be model-informed. Since multiple competing models can explain the same pattern^40^,^41^, it even risks to swamp future research with years of replicating the same, and possibly wrong, pattern analysis (see Table 1). Proposing models with simple, reasonable assumptions and processes can help in identifying the fundamental constituents of the problem, and provide predictions that can be tested and trigger future research and improvements. In the case of human mobility, the random acceleration model proposed here allows for a deeper quantitative understanding, leads to predictions in excellent agreement with data, and brings evidence that the long-standing interpretation with Lévy flights is incorrect. The central idea of our model is the existence of a hierarchical organization of transportation layers with different velocities. The main ingredients that describe the variability of *P*(Δ*r*) among cities (see Supplementary Note 3 and Supplementary Fig. 6) are different travel times (Supplementary Fig. 1), the base speed, the speed gap between layers, and the effective acceleration (Supplementary Fig. 7), which is proportional to the jumping rate to a different layer. An important assumption in this model is that these quantities are constant, but this is not necessarily true. Deviations observed in empirical distributions at the urban level suggest the need for more elaborate velocity models, able to include spatial and temporal inhomogeneities of transportation systems by allowing *v*_0_, *p* and *δv* to vary across cities or during the day.

## Methods

### Private transportation data

We compute spatial displacements and travel times for private car transportation from a database of GPS measures sampling the trajectories of private vehicles in the whole Italy during the month of May 2011. The data are collected for insurance reasons by a device installed on the vehicles and are mainly related to private vehicles, since taxi cabs or delivery companies use their own GPS systems and do not contribute to it. A small percentage of vehicles belongs to private companies and are used for professional reasons. This database includes ≈2% of the vehicles registered in Italy, containing a total of 128,363,000 trips performed by 779,000 vehicles. Records contain information about the engine starts and stops, and travel times include the time spent looking for parking. We introduce a lower threshold of 5 min in the elapsed time to define the end of a trip and to distinguish real stops related to an activity. When the quality of the satellite signal is good, we have an average spatial accuracy of the order of 10 m, but during a trip, the error fluctuations may increase up to 30 m or more^54^. The error in temporal resolution is negligible.

We have applied correction and filtering procedures to exclude from our analysis data affected by systematic errors. Approximately 10% of the data were discarded for this reason. When the engine is switched on or when the vehicle is parked inside a building, there are errors due to signal loss. In such cases, we use the redundant information given by the previous stopping point to correct 20% of the data. When the engine was off for <30 s, the subsequent trajectory is always considered as a continuation of the same trip, except if the vehicle is going back towards the origin of the previous trajectory segment.

For privacy reasons, the drivers' city of residence is unknown. Therefore, it has been necessary to associate to each car an urban area using available information. We do that by identifying a driver as living in a certain city if the most part of its parking time is spent in the corresponding municipality area. For each driver we then consider all trips both within and outside this ‘residence' urban area.

### Measuring the free-flow speed

Each trajectory is sampled at a spatial scale at most equal to 2 km or, in highways, at a time scale of 30 s. Using trajectory reconstructions^55^,^56^, we estimate for each road the average speed for all the trajectories passing through that road. Using the distribution of the travel speed, the free-flow speed of a road is defined as the speed corresponding to the 85% of percentile. As a matter of fact, the free-flow speed corresponds to trajectories carried out during the night and the choice of 85% of percentile takes into account the individual heterogeneity: in many cases there are individuals driving at a speed much larger than the average and that are neglected in the computation of an average free-flow speed.

In Fig. 3a, we have computed the free-flow velocity for the used roads in each trajectory and for different travel time durations (0.5±0.1 h, 1.0±0.1 h and 1.5±0.1 h).

### Details of the model

The model proposed here assumes that urban mobility is performed at a baseline speed of *v*_0_≈18 km h^−1^. Very short trips hardly reach the base speed *v*_0_ that we impose in our model. For this reason, in all the measures proposed in this paper, we considered only trips longer than 1 km and with duration larger than 5 min.

The upper bound of 130 km h^−1^ limits the number of jumps to *k*=(130 km h^−1^−*v*_0_)/*δv*≈3. We have a jumping rate of order *p*′≈1 jump per hour and we expect significant deviations from our prediction for trips longer than 4 h. For such long trips, one cannot in principle approximate the area below the step function in Fig. 3b with a triangle. Indeed, the Poisson fit in Fig. 4 overestimates the frequency of trips faster than 110 km h^−1^ for *t*=120 and 180 min. In Supplementary methods, we show that if the acceleration kicks are limited by a finite number of layers, the speed grows linearly for small times and saturates to a finite limiting speed (see Supplementary Fig. 8). This saturation can be clearly observed at national level (see Fig. 2) and for five out of six of the cities represented in Supplementary Fig. 7.

### Fitting the model parameters

The parameters are computed according to the following procedure. First, we estimate the value of *v*_0_ from a linear fit of the curve 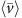
(*t*) (Fig. 2 and Supplementary Fig. 7) in the interval [0, 2] h. Then, the study of the surface generated by the curve 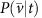 for different durations *t* is limited to the speed interval [*v*_0_, 130] (in km h^−1^) and the time interval [2.5, 182.5] min. The values of speeds are binned at integer values in km h^−1^, whereas trip durations are binned at intervals of 5 min (the centres of the bins fall at *t*=5, 10, 15, …, 180 min). Finally, we estimate the parameters *p*′ and *δv*′ of equation (4) by minimizing the sum of the square errors for all curves (for all *t* bins) simultaneously. In Fig. 4 we show the fits of six sections of the surface. The last parameter needed is the average travel time 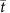 and has been measured separately for each city (see Supplementary Fig. 1). To predict consistently the curve *P* (Δ*r*), the value 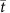
=0.30 h used in Fig. 5 and Supplementary Fig. 6 refers to the trips considered only (Δ*r*>1 km and *t*>5 min).

### Data availability

Anonymized displacement lengths and travel times for the private cars' data are available from the authors upon request.

## Additional information

**How to cite this article:** Gallotti, R. *et al*. A stochastic model of randomly accelerated walkers for human mobility. *Nat. Commun.* 7:12600 doi: 10.1038/ncomms12600 (2016).

## Supplementary Material

Supplementary Information Supplementary Figures 1-8, Supplementary Notes 1-3, Supplementary Methods and Supplementary References.

## Figures and Tables

**Table 1:** Parameter values for the fit of the displacement distribution with a truncated power law found in previous studies.

**Data source**	**Trajectories**	***β***	***κ***	**Δ*****r***_**0**_
Dollar bills^33^	464 K	1.59	∞	0
Mobile phones^11^	100 K	1.75	400 km	1.5 km
Mobile phones^11^	206	1.75	80 km	1.5 km
Mobile phones^12^	3 M	1.55	100 km	0
Location sharing^14^	220 K	1.88	∞	0
GPS tracks^27^	101	[1.16,1.82]	∞	0
Location sharing^15^	900 K	1.50	∞	2.87 km
Location sharing^15^	900 K	4.67	∞	18.42 km
Taxis^19^	12 K	0	4.29 km	—
Taxis^20^	7 K	1.2	10 km	0.31 km
Mobile phones^13^	3 M	0	[2, 5.8] km	—
Travel diaries^9^	230	1.05	50 km	0
Tweets^17^	13 M	1.62	∞	0
Location sharing^16^	521 K	0	300 km	—
Taxis^21^	34 K	0	[2, 4.6] km	—
Taxis^23^	1,100	[0.50, 1.17]	[4.5, 6.5] km	0

This list includes studies on different data sources and spatial or temporal scales. Only fits consistent with the function 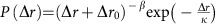 proposed in ref. 11 are presented here. The case *κ*=∞ is associated to non-truncated power laws, while *β*=0 to exponential distributions. When Δ*r*_0_=0 this parameter was omitted in the fit, and we set *β*=0 when this value is not defined. Further studies propose: (i) a polynomial form close to an exponential behaviour for private cars^24^; (ii) two different behaviours for urban and inter-urban trajectories for cars and taxis^22^,^25^; and (iii) a lognormal distribution for individual GPS tracks^28^.
