# Supplementary material for: A stochastic model of randomly accelerated walkers for human mobility
Source: Nat Commun. 2016 Aug 30;7:12600. doi: 10.1038/ncomms12600 (PMC5013551; doi:10.1038/ncomms12600)
Supplement: Supplementary Information — Supplementary Figures 1-8, Supplementary Notes 1-3, Supplementary Methods and Supplementary References. [file ncomms12600-s1.pdf]

Supplementary Figures

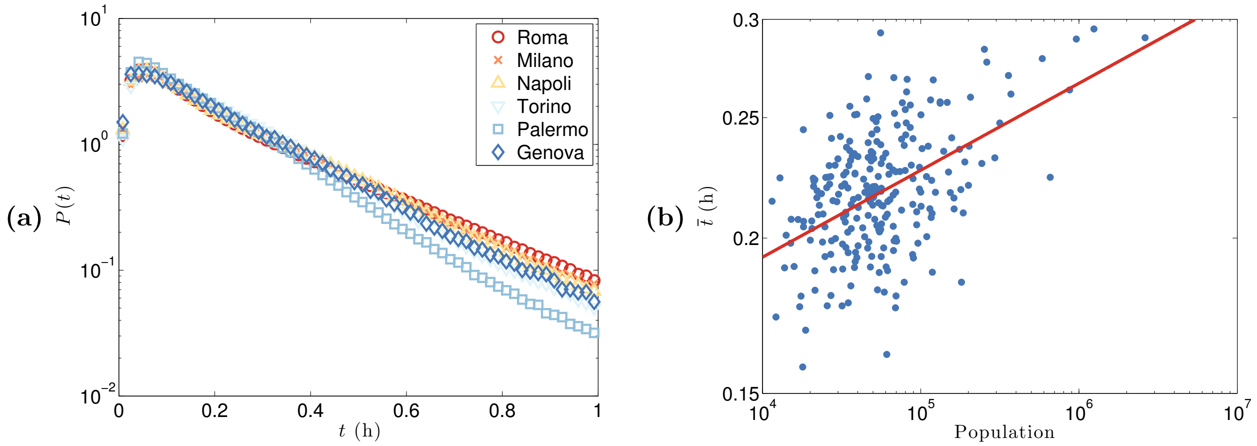

Supplementary Figure 1: **Variation of the average travel-time of private cars across Italian cities.** **(a)** In contrast with the data of Fig. 1, where travel-times of private cars were normalized, here we display the differences between the distributions for the 6 largest cities in Italy. The tail appears to be exponential for travel-times shorter than the classical value of daily travel-time budget of 1 hour [1, 2]. **(b)** We correlate the average travel-time with city populations P. We consider cities with at last 500 drivers that are monitored. The average travel-time (computed here over all trips in a given city) is in the approximate range $[9, 18]$ minutes (with a Spearman correlation $r = 0.40$). The solid line represents a power law fit of the form $\bar{t} \propto P^{\gamma}$ with $\gamma = 0.07 \pm 0.02$.

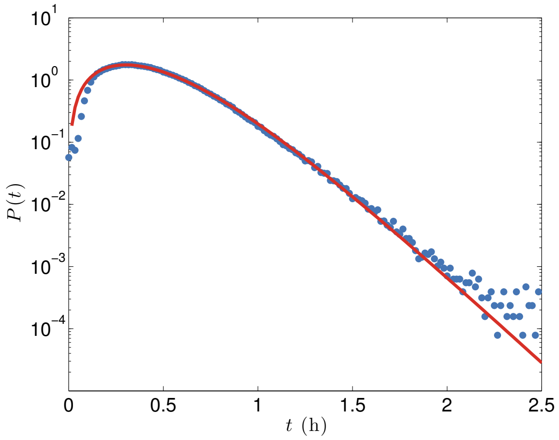

Supplementary Figure 2: **Travel-times distribution for public transportation.** Travel-times extracted from a 5% sample of all Oyster card journeys performed in a week during November 2009 on bus, Tube, Docklands Light Railway (DLR) and London Overground. Data available at https://api-portal.tfl.gov.uk/docs (accessed 3/9/2015). The probability distribution is fitted with the curve $P(t) \propto (1 - \exp(-t/c)) \exp\{-t/b - c\exp(-t/c)/b\}$, which characterizes a duration model with two time-scales [2]. The parameters $c = 36 \pm 2$ minutes and $b = 9.3 \pm 0.3$ minutes show that the average travel-time given by the exponential tail is of order of 9 minutes, but that the exponential behavior is observed for times larger than ≈ 36 minutes.

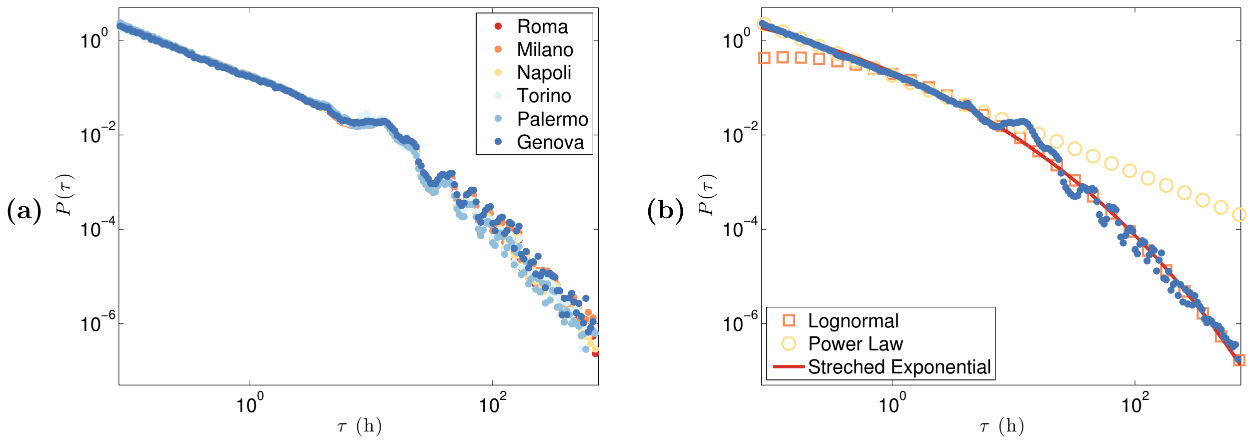

Supplementary Figure 3: **Rest times distribution of private cars.** **(a)** The distributions show little variation among the six largest cities in Italy. **(b)** Considering all the individuals across the whole Italy, we observe that the distribution seems to follow a power law $P(\tau) \propto \tau^{-\eta}$ with $\eta = 1.03 \pm 0.01$, valid for pauses shorter than 4 hours (yellow circles), which represents 74% of the pauses. A lognormal distribution $\ln \mathcal{N}(\mu, \sigma)$ with $\mu = 1.6 \pm 0.6$h and $\sigma = 1.6 \pm 0.1$h could also be proposed to characterize the tail for stops longer than 1 hour (orange squares). A stretched exponential: $P(\tau) \propto \exp(-(\tau/\tau_0)^{\beta})$ with $\beta = 0.19 \pm 0.01$ and $\tau_0 = 10 \pm 5 \cdot 10^{-5}$h successfully fits the whole curve over all the orders of magnitude observed here (red solid line).

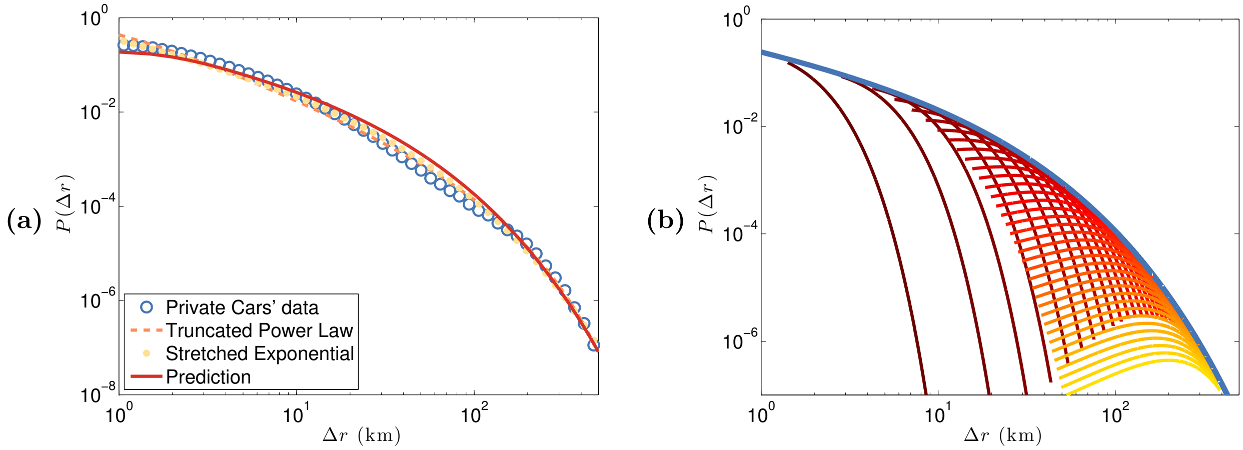

Supplementary Figure 4: **Distribution of displacements. (a)** Similarly to Fig. 5, we represent with blue circles the aggregated empirical distribution $P(\Delta r)$ for all the $\approx 780,000$ cars in our dataset. The orange dashed line represents a fit with a truncated power law of the form $P(\Delta r) \propto (\Delta r + \Delta r_0)^{-\beta} \exp(-\Delta r/\kappa)$ with $\beta = 1.67$, $\kappa = 84.1$ km, $\Delta r_0 = 0.63$ km. The coefficients of the fit are close to those found for mobile phone data in the US [3]. The yellow dots represents the fit with the stretched exponential Eq. (2) of the main text (with $C = 0.49$ km$^{-0.5}$) associated to uncorrelated accelerations model. The red solid line shows the prediction based on our model Eq. (5) (see Fig. 5). **(b)** Visual representation of the integral in Eq. (5). The $P(\Delta r)$ (blue line) is obtained as the superimposition of Poisson distributions of Eq. (4) with different $\lambda = p' t$. We display the distribution for $t = 5, 10, 15, \ldots, 180$ minutes with a colorcode going from red to yellow. The domain of these curves is limited by speeds between v_0 and 130km/h. The area under each curve is decreasing with time as $\propto e^{-t/\bar{t}}$.

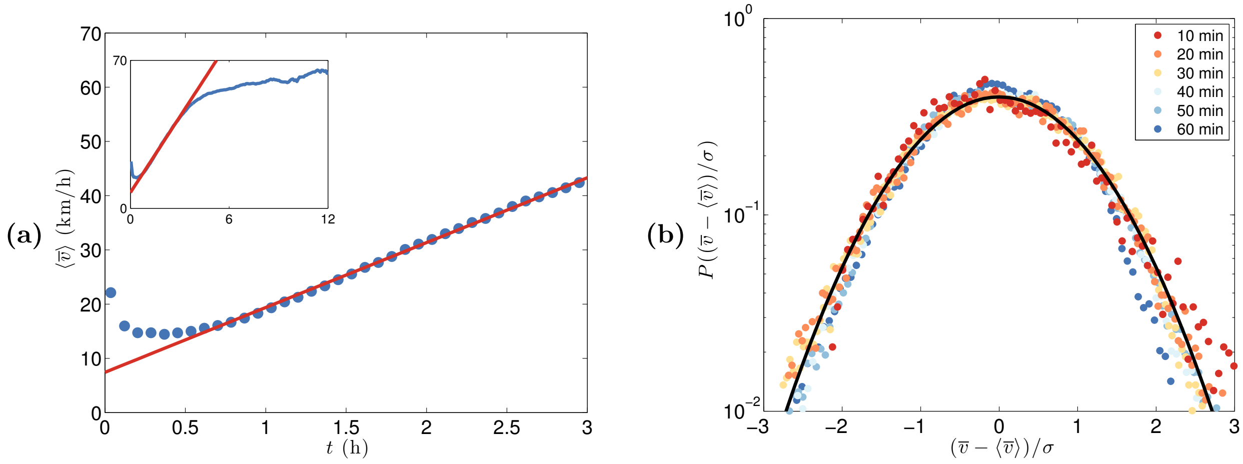

Supplementary Figure 5: **Acceleration of public transportation.** **(a)** For public transportation trajectories (linking all possible origins and destinations in Great Britain [4], see Supplementary Note 2), we also observe a uniform acceleration for trips of duration between 30 minutes and 3.5 hours. The solid red line represents the fit with $v_0 = 7.4 \pm 0.4$ km/h and $a = 12.0 \pm 0.2$ km/h^2. In this case, very short trips are faster thanks to the likely absence of connections. **(b)** Conditioned speed distribution for trajectories originated in Charing Cross in London. If we restrict ourselves to trips of duration shorter than 1 hour, the distribution is Gaussian.

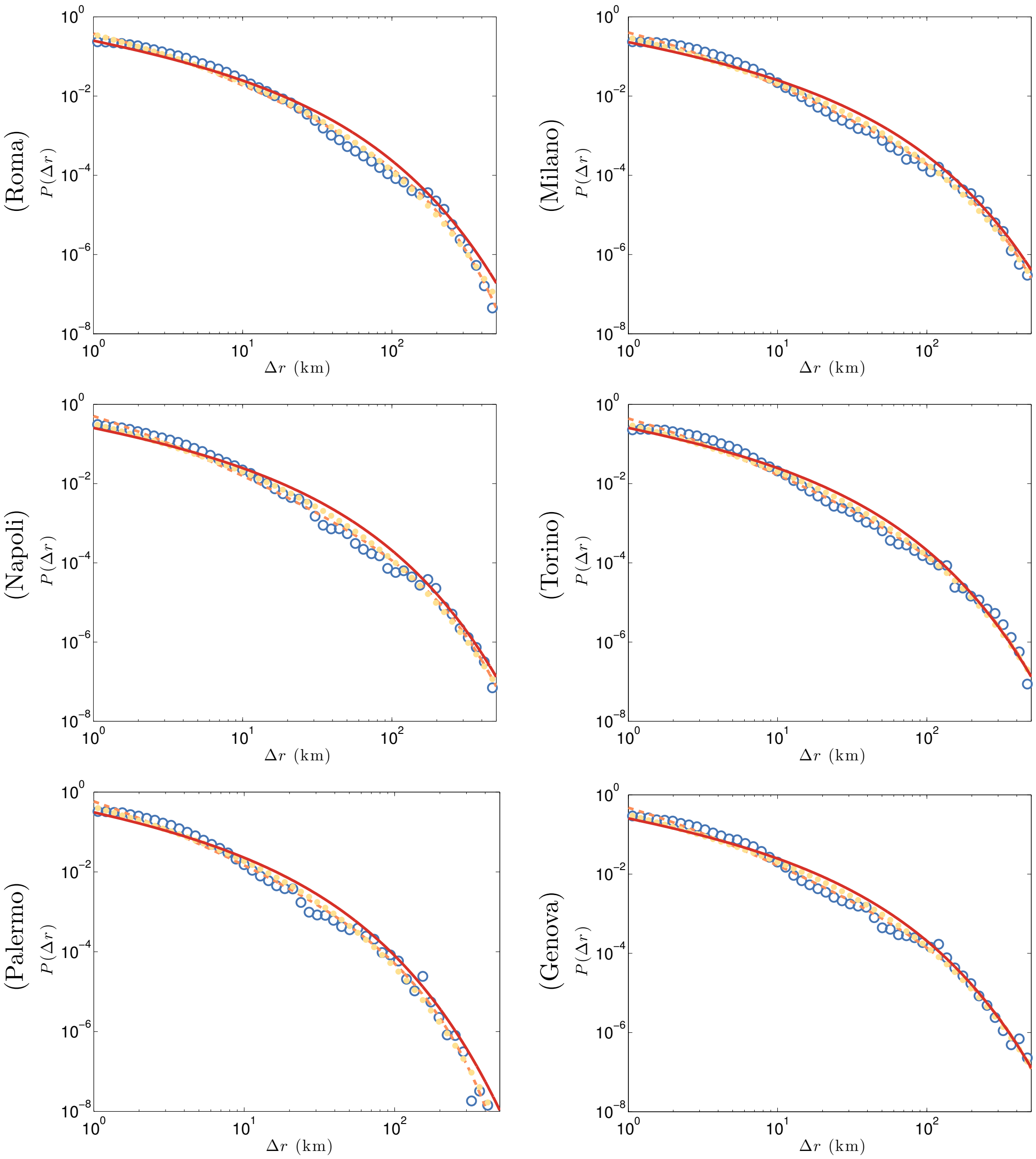

Supplementary Figure 6: **Displacement distribution for the 6 largest Italian cities.** Similarly to Supplementary Fig. 4 (a), we display here the prediction of our model for the distribution $P(\Delta r)$ in the six largest Italian cities. The blue circles represent the empirical distribution. The orange dashed line represents the fit with a truncated power law [3] and the yellow dots the fit with a stretched exponential (Eq. (2)). The red solid line is the prediction Eq. (5). The fit values for the cities are in the ranges $p' = [0.97, 1.81]$ jumps/h, $\delta v' = [13.2, 18.5]$ km/h, $\bar{t} = [0.30, 0.37]$ h while $v_0 = 16.5$ km/h.

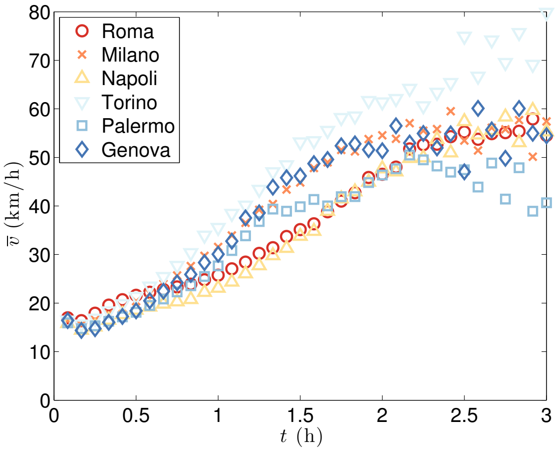

Supplementary Figure 7: **Average speed growth versus travel-time in the 6 largest Italian cities.** The speed growth for single cities is less uniform than in the aggregate case of Fig. 2. This is probably related to inhomogeneities of the road network and geographical characteristics of the region around each city. All curves share a similar value of $\overline{v} = 16.5 \pm 0.5$ km/h for $t = 5$ min, therefore this constant value has been selected as v_0 for the prediction proposed in Supplementary Fig. 6.

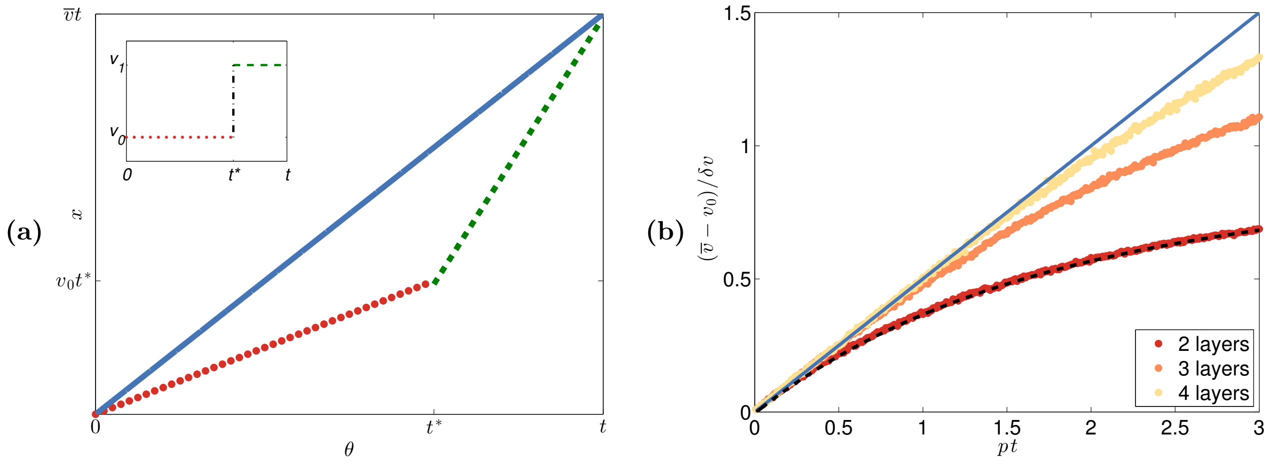

Supplementary Figure 8: **Average speed in a one-dimensional multilayer hierarchical transportation infrastructure.** **(a)** The dotted line in the plane (θ, x) represents a trajectory where a time t^* is spent on the base layer with speed v_0 (red dots) and a time $t - t^*$ is spent travelling at speed v_1 on the fast layer (green dashed line). The average speed $\overline{v}$ is simply the weighted average of the two speeds (blue solid line). **(b)** A numerical simulation confirms the result of Eq. (S4) (black dashed line, see Supplementary Methods) for the case of two layers. We also simulated the case of more than two layers where the linear growth regime $\overline{v} \approx v_0 + \frac{1}{2}p(v_1 - v_0)$ (blue solid line) extends beyond $pt \ll 1$.

Supplementary Note 1

The individual mobility is essentially composed by two different phases, travels and rests, both having a finite duration.

Trip duration

Studying times instead of distances allows to exclude the variability due to the different speeds, which may contribute to the perceived difference in travel costs. When travel costs are homogeneous, their distribution is expected to be exponential [5]. Similarly to what is observed for total daily travel-times [6, 7, 2], the exponential distribution appears in a variety of context such as the difference between independent events or maximizing the entropy at fixed average. We observe that the distribution of cars' travel-times is indeed characterized by an exponential tail (Fig. 1 and Supplementary Fig. 1 (a)).

For public transportation systems, we also observe a rapidly decreasing tail for the travel-times between metro stations in London evaluated from the oyster card ticketing system. (See [8] and Supplementary Fig. 2).

Travel-times for cars however depend on the city population [9]: the largest the city, and the worse congestion effects. This is confirmed by our results: if we bin the drivers according to their city of residence (see Methods), we find that the average travel-time in different cities falls in the range $[9 \text{ min}, 18 \text{ min}]$, with a growth correlated with population $\bar{t} \propto P^{\mu}$, where $\mu = 0.07 \pm 0.02$ (Supplementary Fig. 1 (b)). As a consequence, the travel time distribution $P(t)$ differs among cities (Supplementary Fig. 1 (a)), but if we rescale the trip duration by its average value, we observe in Fig. 1 that the distribution $P(t/\bar{t})$ appears as universal across different cities and is well approximated by

$$P(t) = \frac{1}{\bar{t}} \mathrm{e}^{-t/\bar{t}} \tag{S1}$$

The average value $\bar{t}$ contains then all the information needed for describing cars' travel-times in a particular city.

Rest duration

The rest time τ is another fundamental aspect in modeling human mobility [10]. The characteristics of the distribution $P(\tau)$ strongly influence entropy [11], and therefore the predictability of individual mobility patterns [12]. We observe that $P(\tau)$ does not display remarkable differences among cities for stops within 24 hours (see Supplementary Fig. 3 (a)). Consistently with the delay time distribution in email communications [13, 14], this distribution is close to a power law with exponent $\simeq -1$ [7, 11] (for times shorter than 12 hours). A lognormal fit for the tail seems also reasonable [15]. In Supplementary Fig. 3 (b), we show the best fits with these functions, while we also propose a fit with a stretched exponential that, although it represents a good fit for all times between 5 minutes and 30 days, lacks any theoretical explanation.

Supplementary Note 2

Dependence of speeds over time in public transportation.

We estimate speeds for public transportation trips from an open dataset providing a complete snapshot of the multilayer temporal network of public transportation in Great Britain in October 2010 [4]. This dataset assumes a waiting time before a flight of 2 hours, a waiting time after a flight of 30 minutes and, where not explicitly defined by the transportation agencies, a walking distance of 250 m. The starting time in our analysis of a week is Monday, 8am. Starting from that time, we consider for all trips from a node i to a node j, the time spent in transportation after departing from the node i in any direction. The shortest time-respecting path [16] is then identified with a Dijskstra algorithm, where also the time spent walking (at 5 km/h) between two adjacent stops of any modes of transport and the time spent waiting at the connection is integrated over the total travel-time. The euclidean distance between origin and destination is then used for estimating the speed $\overline{v}$.

Both average values and the distribution of Supplementary Fig. 5 are not computed on real flows, which are not available with the same spatial extension and definition of the dataset. In this study, we implicitly assumed a uniform travel demand by including all possible origin-destination pairs.

We observe a similar linear growth for the average speeds of trajectories in the public transportation system (Supplementary Fig. 5 (a)). In this case we do not have actual individual trajectories but if we assume that there is a uniform travel demand and that travellers make the shortest time-respecting path between origin and destination [16], we can estimate the average speed $\langle\overline{v}\rangle$. Short trips, of less than 30 minutes, tend to be faster thanks to the likely absence of time-consuming connections. For $t \in [0.5, 3.5]$ hours, the growth is again linear but both base speed $v_0 = 7.4 \pm 0.4$ km/h and acceleration $a = 12.0 \pm 0.2$ km/h^2 are smaller than in the case of private transportation. In Supplementary Fig. 5 (b) we show that for urban trajectories in London ($t < 1$h from Charing Cross) the distribution $P(\overline{v})$ is universal and Gaussian-like. The difference between the exponential shape of $P(\overline{v})$ for private transportation and the Gaussian-like distribution for public transportation might originate in the fact that in public transportation $\overline{v}$ of a trajectory is obtained as the weighted average of the speed associated to each edge of the transportation network (plus the effect of waiting time), leading to a normal distribution according to the central limit theorem [17]. In principle, public transports move according to a deterministic dynamics and arrival times at stations is fixed. However, fluctuations in the average speed of a trip lead to small delays at different stations and when summed together – for a trip with connections – lead to a Gaussian distribution.

Supplementary Note 3

Variability of $P(\Delta r)$ among cities

We note that for single cities there are of course local differences and that there are deviations from the linear trend for the average speed of trips. These are probably due to the inhomogeneities of the structure of the road networks. The linear trend is however a good fit, as can be seen in Supplementary Fig. 7. Moreover, all cities seem to have a similar average speed of ≈ 16.5 km/h at $t = 5$ minutes. We use this value as v_0 and estimate, for each of the 6 cities, p' and $\delta v'$ by fitting the conditional probability $P(\overline{v}|t)$ between v_0 and 130 km/h. Finally, we also compute the average travel-time $\bar{t}$ for each city. This allows us to use Eq. (5) to predict the exact shape of $P(\Delta r)$ for trajectories of drivers living in different cities. In Supplementary Fig. 6, we show that this prediction (red solid line) is correct for all cities, and it can be compared with the a-posteriori fit with a truncated power law (orange dash line) or the stretched exponential (Eq. (2), yellow dots).

Supplementary Methods

Accelerated walker with a limited number of layers

We consider the one dimensional case (the extension to 2d is simple) for a transportation network with only 2 possible layers: L_0 and L_1 corresponding to different travel speeds. On the layer L_0, individuals travel with speed v_0, whereas on L_1 they are traveling faster at speed $v_1 = v_0 + dv$. An individual starts its trip of duration t in L_0 and has a probability per unit time p to jump to layer L_1 and to increase its speed. Being a Poisson process, the probability to jump at time t^* is then given by

$$P(t^* = t) = p\mathrm{e}^{-pt} \tag{S2}$$

and the position at time $t\theta$ of the traveller is

$$x(t) = v_0\theta H(t^* - t) + H(t - t^*)\left[v_0 t^* + v_1(t - t^*)\right] \tag{S3}$$

where $H(x)$ is the Heaviside function (see Supplementary Fig. 8 (a)). By averaging the position over t^*, we obtain for the average speed $\overline{v} = \overline{x}/t$ the following simple expression

$$\overline{v}(t) = v_1 + (v_0 - v_1)\frac{1 - e^{-pt}}{pt} \tag{S4}$$

(the bar $^-$ denotes the average over different trips). For the limiting case $pt \ll 1$ the average speed grows linearly from the base value v_0: $\overline{v}(pt \ll 1) \approx v_0 + \frac{1}{2}pt(v_1 - v_0)$. This result is consistent with Eq. (3), up to the change $t \to t/2$ due to the fact that we consider the acceleration regime only. For $pt \gg 1$ the average speed converges asymptotically to v_1: $\overline{v}(pt \gg 1) \approx v_1 - (v_1 - v_0)/pt$. Under certain conditions this simple model thus recovers the linear growth of speed with the duration of the trip, and also the tendency to reach a limiting speed observed in Fig. 2.

If we have more than two layers with the same jumping probability and constant speed gap $v_{n+1} - v_n = \delta v$ between consecutive layers L_n and L_{n+1}, the constant acceleration regime is extended up to $pt > 1$ (see Supplementary Fig. 8 (b)). This result suggests that a multilayer hierarchical transportation infrastructure can explain the constant acceleration observed in both public and private transportation systems. From this model, we can also estimate the base speed v_0 with the value of the intercept in Fig. 2, and Supplementary Figures 5 and 7, whereas the acceleration a is expected to be proportional to the probability of jump to faster layers p and to the gap between layers δv.

Saddle-point analysis of $P(\Delta r)$.

Random velocity model

From Eq. (1), the average speed distribution is a Gaussian distribution

$$P(|\overline{v}|) = \frac{2}{\sqrt{2\pi D t}}\mathrm{e}^{-\frac{|\overline{v}|^2}{2Dt}} \qquad \overline{v} > 0 \tag{S5}$$

and the trip duration is exponential

$$P(t) = \frac{1}{\overline{t}}\mathrm{e}^{-t/\overline{t}} \tag{S6}$$

Writing $\Delta r = \overline{v}t$ we get the following expression for $P(\Delta r)$

$$P(\Delta r) = \int_0^\infty \int_0^\infty \frac{\mathrm{d}t}{\bar{t}} \frac{2\mathrm{d}|\overline{v}|}{\sqrt{2\pi D t}} \delta(\Delta r - |\overline{v}|t) \mathrm{e}^{-\frac{|\overline{v}|^2}{2Dt} - \frac{t}{\bar{t}}} \tag{S7}$$

This can be rewritten as

$$P(\Delta r) = \sqrt{\frac{2}{\pi D}} \int_0^\infty \frac{\mathrm{d}t}{t^{3/2}\bar{t}} \mathrm{e}^{-F(\Delta r, t)} \tag{S8}$$

where

$$F(\Delta r, t) = \frac{1}{2} \frac{\Delta r^2}{D t^3} + \frac{t}{\bar{t}} \tag{S9}$$

At large r and t this integral is governed by the saddle point defined by $dF/dt = 0$ which then leads to

$$P(\Delta r) \sim \frac{1}{\Delta r^\gamma} \mathrm{e}^{-C \Delta r^\delta} \tag{S10}$$

with $\gamma = 3/4$ and $\delta = 1/2$.

Random kicks model

The expression for $P(\Delta r)$ can be rewritten as

$$P(\Delta r) = \int \frac{\mathrm{d}t}{t\bar{t}\delta v'} \mathrm{e}^{-F(\Delta r, t)} \tag{S11}$$

where

$$F(r,t) = \left(p' + \frac{1}{\bar{t}}\right) - \frac{r - v_0 t}{\delta v' t} \log(p't) + \log \Gamma \left(1 + \frac{r - v_0 t}{\delta v' t}\right) \tag{S12}$$

At large r and t, this integral is governed by the saddle point defined by $dF/dt = 0$ which reads

$$0 = p' + \frac{1}{\bar{t}} - \frac{1}{\delta v'} \left(-\frac{r}{t^2} + \frac{r - v_0 t}{t^2}\right) - \frac{r}{\delta v' t^2} \Psi \left(1 + \frac{r - v_0 t}{\delta v' t}\right) \tag{S13}$$

where Ψ is the Digamma function. We then obtain for large times, the scaling $r \sim t^2$, which implies the following behavior

$$P(\Delta r) \sim \Delta r^{-\gamma} \mathrm{e}^{-\Delta r^{1/2}} \tag{S14}$$

where the exponent γ here depends in general on the parameters v_0 and $\delta v'$. We note that this behavior will also be recovered even if we have Gaussian velocity distribution as it is observed for public transportation.

Supplementary References

[1] Metz, D. The myth of travel time saving. *Transp Rev* **28**, 321–336 (2008).

[2] Gallotti, R., Bazzani, A. & Rambaldi, S. Understanding the variability of daily travel-time expenditures using GPS trajectory data. *EPJ Data Science* **4**, 1–14 (2015).

[3] González, M.C., Hidalgo, C.A. & Barabási, A.-L. Understanding individual human mobility patterns. *Nature* **453**, 779–782 (2008).

[4] Gallotti, R. & Barthelemy, M. The multilayer temporal network of public transport in Great Britain. *Sci Data* **2**, 140056 (2015).

[5] Yan, X.-Y., Han, X.-P., Wang, B.-H. & Zhou, T. Diversity of individual mobility patterns and emergence of aggregated scaling laws. *Sci Rep* **3**, 2678 (2013).

[6] Kölbl, R., Helbing, D. Energy laws in human travel behaviour. *New J Phys* **5**, 48.1–48.12 (2003).

[7] Gallotti, R., Bazzani, A. & Rambaldi, S. Toward a statistical physics of human mobility. *Int J Mod Phys C* **23**, 1250061 (2012).

[8] Roth, C., Kang, S. M., Batty, M. & Barthelemy, M. Structure of urban movements: polycentric activity and entangled hierarchical flows. *PLoS ONE* **6**, e15923 (2011).

[9] Louf, R. & Barthelemy, M. How congestion shapes cities: from mobility patterns to scaling. *Sci Rep* **4**, 5561 (2014).

[10] Song, C., Koren, T., Wang, P. & Barabási, A.-L. Modelling the scaling properties of human mobility. *Nature Phys* **6**, 818–823 (2010).

[11] Gallotti, R., Bazzani, A., Degli Esposti, M. & Rambaldi, S. Entropic measures of individual mobility patterns. *J Stat Mech* **2013**, P10022 (2013).

[12] Song, C., Qu, Z., Blumm, N. & Barabasi, A.-L. Limits of predictability in human mobility. *Science* **327**, 1018 (2010).

[13] Barabási, A.-L. The origin of bursts and heavy tails in human dynamics. *Nature* **435**, 207–211 (2005).

[14] Malmgren, R. D., Stouffer, D. B., Motter, A. E. & Amaral, L. A. N. A Poissonian explanation for heavy tails in e-mail communication. *Proc Natl Acad Sci USA* **105**, 18153–18158 (2008).

[15] Stouffer, D. B., Malmgren, R. D. & Amaral L. A. N. Log-normal statistics in e-mail communication patterns. arXiv:physics/060527 (2006).

[16] Gallotti, R. & Barthelemy, M. Anatomy and efficiency of urban multimodal mobility. *Sci Rep* **4**, 6911 (2014).

[17] Strano, E., Shay, S., Dobson, S. & Barthelemy, M. Multiplex networks in metropolitan areas: generic features and local effects. *J R Soc Interface* **12**, 20150651 (2015).
